# Carbon Dioxide Solubility in Nonionic Deep Eutectic Solvents Containing Phenolic Alcohols

**DOI:** 10.3389/fchem.2022.864663

**Published:** 2022-03-22

**Authors:** Ahmad Alhadid, Javid Safarov, Liudmila Mokrushina, Karsten Müller, Mirjana Minceva

**Affiliations:** ^1^ Biothermodynamics, TUM School of Life Sciences, Technical University of Munich (TUM), Freising, Germany; ^2^ Institute of Technical Thermodynamics, University of Rostock, Rostock, Germany; ^3^ Institute of Separation Science and Technology, Friedrich-Alexander-Universität Erlangen-Nürnberg (FAU), Erlangen, Germany

**Keywords:** CO_2_ capture, COSMO-RS, ionic liquids, hydrophobic deep eutectic solvents, green solvents

## Abstract

Deep eutectic solvents (DES) are a new class of green solvents that have shown unique properties in several process applications. This study evaluates nonionic DES containing phenolic alcohols as solvents for carbon dioxide (CO_2_) capture applications. Potential phenolic alcohols and the molar ratio between DES constituents were preselected for experimental investigations based on the conductor-like screening model for realistic solvation (COSMO-RS). CO_2_ solubility was experimentally determined in two different DES, namely, L-menthol/thymol in 1:2 molar ratio and thymol/2,6-xylenol in 1:1 molar ratio, at various temperatures and pressures. CO_2_ solubility in the studied systems was higher than that reported in the literature for ionic DES and ionic liquids. This study demonstrates that nonionic DES containing phenolic alcohols can be excellent, inexpensive, and simple solvents for CO_2_ capture.

## Introduction

An increasing relevance has developed in removing carbon dioxide (CO_2_) from gas mixtures, such as flue gas or biogas. Absorption in liquid solvents, in addition to membrane and absorption-based processes, is a major technology in this field. For many years, aqueous solutions of amines have been used for CO_2_ absorption. However, they suffer from some drawbacks, such as high vapor pressure, which causes evaporation during solvent regeneration. Recently, ionic liquids (IL) have drawn attention for CO_2_ capture application because of their negligible vapor pressure (Albo et al., 2010; Shiflett et al., 2010). Furthermore, the properties of IL can be tailored to the requirements of the specific application by combining different cations and anions (Jork et al., 2005). Although the hygroscopicity of IL can be overcome by using polymerized IL to prepare membranes for CO_2_ capture applications (Kammakakam et al., 2020; Galiano et al., 2021), the issues of IL cost, instability, and purity remain (Sowmiah et al., 2009).

Nevertheless, owing to the aforementioned problems of IL, researchers have focused on an alternative solvent class that has some similarities to IL, while avoiding some of their drawbacks. Deep eutectic solvents (DES) are eutectic mixtures with a large depression in the eutectic temperature obtained by mixing a hydrogen bond acceptor and donor. DES are a new class of designer solvents, which can also be prepared by simple mixing of natural and nontoxic components, usually referred to as Natural DES (NADES) (Smith et al., 2014; van Osch et al., 2020). Similar to IL, physicochemical properties of DES can be tuned by selecting their constituents and additionally molar ratios of those. Moreover, DES are easier and less expensive to prepare compared to IL. Therefore, more attention is directed to their use in several process applications, for example, in liquid–liquid chromatography (Roehrer et al., 2016; Bezold and Minceva, 2019; Cai and Qiu, 2019), extraction of bioactive compounds (Kalhor and Ghandi, 2019; Makoś et al., 2020; Perna et al., 2020; Rodríguez-Llorente et al., 2020; Fernández et al., 2022), and crystallization (Emami and Shayanfar, 2020; Hall et al., 2020; Hamilton et al., 2020; Potticary et al., 2020).

CO_2_ capture is one application that can benefit from DES. Existing studies have demonstrated the potential of DES for CO_2_ capture (Krishnan et al., 2020; Song et al., 2020; Wazeer et al., 2021a; Wazeer et al., 2021b). However, most studies investigating CO_2_ capture in DES proposed using ionic DES, which has the same drawbacks as those related to IL, especially in terms of hygroscopicity. Recently, hydrophobic DES based on natural and inexpensive nonionic constituents has attracted much attention (van Osch et al., 2019). Hydrophobic DES containing L-menthol possess outstanding properties, such as low viscosity and eutectic temperatures, especially when L-menthol is mixed with phenolic alcohols, such as thymol or carvacrol (Alhadid et al., 2020a; Alhadid et al., 2020b; Alhadid et al., 2021a; Alhadid et al., 2021b). Phenolic IL are good solvents for CO_2_ capture applications (Vafaeezadeh et al., 2015). Therefore, hydrophobic DES containing phenolic alcohols are assumed to be promising candidates for CO_2_ capture applications.

Nevertheless, the large pool of substances that can form DES can make selecting the DES constituents challenging. Furthermore, the ratio between the constituents can be tuned, which is an additional degree of freedom during the selection of DES constituents. Therefore, a predictive screening method could noticeably assist in preselecting DES constituents for CO_2_ capture applications. The conductor-like screening model for realistic solvation (COSMO-RS) is a predictive thermodynamic model based on quantum mechanics and statistical thermodynamics (Klamt, 1995; Klamt et al., 1998; Eckert and Klamt, 2002). COSMO-RS successfully provides qualitative predictions for screening IL and ionic DES for gas capture applications (Völkl et al., 2012; Song et al., 2020; Liu et al., 2021; Qin et al., 2021).

This study investigates nonionic DES containing phenolic alcohols as potential solvents for CO_2_ capture. COSMO-RS was used to screen a list of possible DES containing L-menthol and phenolic alcohols to preselect those with the highest CO_2_ solubility. Further, CO_2_ solubility was experimentally investigated for selected DES systems at various temperatures and pressures using an isochoric method.

## Materials and Methods

### Eutectic Mixture Preparation

Pure components (L-menthol, purity ≥99%, Sigma Aldrich; thymol, purity ≥99%, Sigma Aldrich; 2,6-xylenol, purity 99%, Acros Organics), was mixed under continuous stirring and gentle heating until a clear homogenous liquid was formed. The water content of the prepared eutectic mixtures was measured in triplicate using Karl Fischer Coulometer (Hanna Instrument, United States), and the results are shown in Table 1.

**TABLE 1:** Prepared eutectic mixtures, their molar ratio, and water content.

Eutectic mixture	Mole ratio	Water content/ppm
L-menthol/thymol	1:2	144.2 ± 2.1
Thymol/2,6-xylenol	1:1	108.4 ± 6.7

### Carbon Dioxide Solubility Experiments

Before measurements, DES were degassed under vacuum for 48 h at a temperature of *T* = 413.15 K. After degassing, no mass loss was observed for the DES, indicating that there was no change in the stoichiometry between constituents and their negligible volatility under the experimental conditions. CO_2_ from Westfalen AG, Germany, with a purity of 99.995% (quality 4.5), was used without further purification. The experiments were performed using a pressure-drop isochoric method at various temperatures and pressures. The apparatus and operational procedures of solubility measurements are described in detail in previous studies (Safarov et al., 2013; Safarov et al., 2014). For the current measurements, the installation was used without modification. The temperature in the measuring cell was held constant (at *T* = 293.15–323.15 K) with an uncertainty of u(T) = 0.030 K. A pressure transducer with an accuracy of 0.1% was used to measure the pressure of CO_2_ filled in the gas reservoir. The temperature inside the gas reservoir was measured with an uncertainty of u(T) = 0.015 K. The initial amount of CO_2_ in the gas reservoir was determined from its pressure and temperature using the Span and Wagner equation of state (Span and Wagner, 1996).

To determine the concentration of CO_2_ in the solution, liquid and gas densities under experimental temperature and pressure were required. The density of DES was measured using a density meter (Density meter Easy D40, Mettler-Toledo GmbH, Germany), and the results are indicated in Table 2. The mass of CO_2_ in the gas phase was calculated using its density and volume. The gas volume in the cell was found by deducting the liquid volume from the total cell volume. The increase in the liquid volume because of dissolved CO_2_ was neglected (Safarov et al., 2013).

**TABLE 2:** Density of eutectic mixtures measured in this work^a^.

*T*/K	*ρ*/kg m^−3^	
	L-menthol/thymol (1:2)	Thymol/2,6-xylenol (1:1)
293.15	947.6	992.2
303.15	940.0	983.9
313.15	932.5	975.6
323.15	924.8	967.2

a Standard uncertainty *u*(*ρ*) = 0.05 kg m^−3^

### Correlation of the Gas Solubility

Henry’s law for a binary system (liquid + CO_2_) for a non-ideal gas phase can be given as (Prausnitz and Shair, 1961)

$$y_{CO_{2}}\cdot \phi_{{\text{CO}}_{2}}\left(T,p\right)\cdot p=x_{{\text{CO}}_{2}}\cdot H_{{\text{CO}}_{2}}$$ (1)

where $y_{{\text{CO}}_{2}}$ and $x_{{\text{CO}}_{2}}$ are the mole fraction of CO_2_ in the gas and liquid phases, respectively; $\phi_{{\text{CO}}_{2}}$ is the fugacity coefficient of CO_2_ in the gas phase, and $H_{{\text{CO}}_{2}}$ is Henry’s constant. Due to the negligible vapor pressure of the studied eutectic mixtures at studied temperatures (Xin et al., 2021) (see also Supplementary Table S1 in Supplementary Material for calculations), the gas phase can be assumed as pure CO_2_, i.e., $y_{CO_{2}}=1$ (see also Supplementary Table S1 in Supplementary Material for calculations). Henry’s constant can be defined using the following equation.

$$H_{{\text{CO}}_{2}}=\underset{p\to \;0}{\lim}\left[\frac{\phi_{{\text{CO}}_{2}}\left(T,p\right)\cdot p}{x_{{\text{CO}}_{2}}}\right]$$ (2)

### COSMO-RS Calculations

The solubility of CO_2_ in eutectic mixtures was screened a priori by evaluating CO_2_ activity coefficients at infinite dilution. The activity coefficients of CO_2_ at infinite dilution in different pure constituents and eutectic mixtures were calculated using the COSMO-RS model (BIOVIA COSMOtherm X19, Dassault Systèmes) and BP_TZVP_19. ctd parameters. Molecular conformations of components were obtained using BIOVIA COSMOconf 17 (Dassault Systèmes). The geometry optimization and screening charge density were determined by density functional theory calculations using BP86 functional and def-TZVP basis set by Turbomole version 6.6 (TURBOMOLE GmbH).

## Results and Discussion

### Screening With COSMO-RS

**FIGURE 1:**
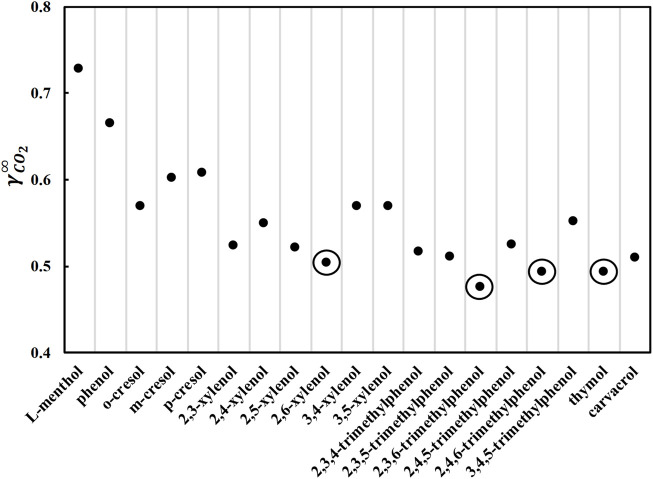
Activity coefficients at infinite dilution of carbon dioxide in pure L-menthol and various phenolic alcohols at 293.15 K calculated by COSMO-RS.

To enable the usage of a DES in CO_2_ capture applications, the DES should 1) be liquid in the appropriate temperature range, i.e., approximately room temperature; and 2) be a good solvent for CO_2_. A number of nonionic DES containing phenolic alcohols can satisfy these two criteria. The phenolic alcohols considered in this study are phenol, methylphenols (cresols), dimethylphenols (xylenols), trimethylphenols, and two natural phenolic terpenes, thymol (2-isopropyl-5-methylphenol) and carvacrol (5-isopropyl-2-methylphenol). Phenolic alcohols with higher molecular weights or dihydroxy benzenes were not considered because they are not expected to form liquid DES at room temperature because of their high melting temperature and enthalpy (Alhadid et al., 2019). When phenolic alcohols are mixed with L-menthol, a significant negative deviation from the ideal behavior is expected (Alhadid et al., 2020b; Alhadid et al., 2021a). The negative deviation from the ideal behavior results in the formation of DES with a sufficiently low melting temperature. Thus, it is desirable to have L-menthol as a DES constituent. First, the activity coefficients of CO_2_ at infinite dilution were calculated using COSMO-RS in pure constituents at 293.15 K because the physicochemical properties of pure constituents influence the physicochemical properties of DES (Alhadid et al., 2021b), and the results are shown in Figure 1. As COSMO-RS is based on quantum mechanical calculations, the calculated activity coefficients implicitly include the atomistic rationalization and quantify the intermolecular interactions between CO_2_ and the constituents in the liquid phase. The low CO_2_ activity coefficient values indicate strong intermolecular interactions between CO_2_ and the constituents and, accordingly, high CO_2_ solubility. The calculated CO_2_ activity coefficients in all phenolic alcohols are lower than in L-menthol (cyclohexyl alcohol) (Figure 1), proving that CO_2_ solubility is relatively high in phenolic alcohols.

Figure 1 shows that the limiting activity coefficients decrease with the addition of methyl groups to phenolic alcohols. The general order of the CO_2_ activity coefficients in phenolic alcohols is phenol > methylphenol (cresol) > dimethylphenol (xylenol) > trimethylphenols. Furthermore, compared to thymol and carvacrol, substituting a methyl group with an isopropyl group, i.e., 2,5-xylenol, decreases CO_2_ limiting activity coefficients. By comparing different isomers, the limiting activity coefficients are lower in isomers with methyl groups at position 2, i.e., close to the hydroxyl group. Furthermore, the lowest values of CO_2_ activity coefficients are observed in dimethyl and trimethyl isomers with a methyl group on the two and six positions, respectively. The CO_2_ activity coefficient in thymol is lower than that in carvacrol, as the isopropyl group is nearer to the hydroxyl group in thymol than to carvacrol. Therefore, the structure of the phenolic alcohol influences CO_2_ solubility. The four marked phenolic alcohols with the lowest limiting activity coefficient values in CO_2_ were considered potential DES constituents for further screening.

**FIGURE 2:**
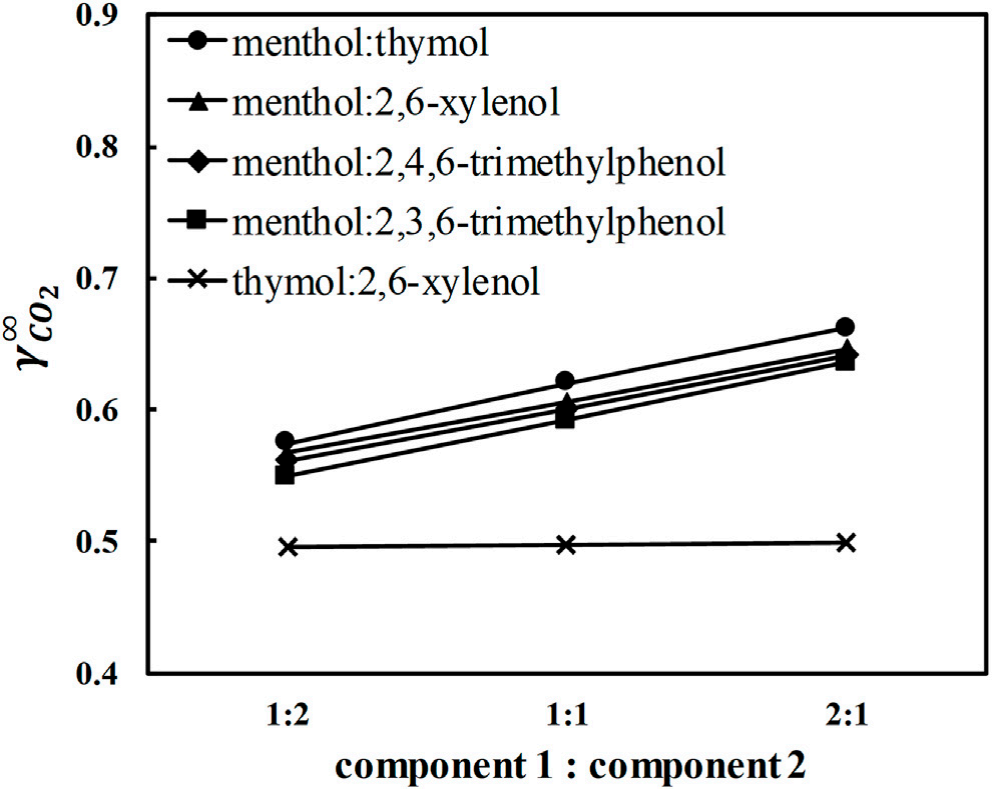
Activity coefficients at infinite dilution of carbon dioxide in selected eutectic mixtures at 293.15 K calculated by COSMO-RS.

Further, potential DES containing L-menthol with 2,6-xylenol, thymol, 2,3,6-trimethylphenol, and 2,4,6-trimethylphenol were screened using COSMO-RS. The calculated activity coefficients of CO_2_ at infinite dilution in five different DES and ratios between the constituents at 293.15 K are shown in Figure 2. CO_2_ activity coefficients in L-menthol-based DES at any molar ratio are in the order L-menthol:thymol (MTH) > L-menthol:2,6-xylenol (M26X) > L-menthol:2,4,6-trimethylphenol (M246) > L-menthol:2,3,6-trimethylphenol (M236) (Figure 2), which is consistent with the order of CO_2_ activity coefficients in the pure phenolic alcohols present in the DES (see Figure 1). Moreover, increasing the molar ratio of the phenolic alcohol to L-menthol decreases CO_2_ activity coefficients. Therefore, it is logical to select L-menthol-based DES containing trimethylphenols in a 1:2 ratio between the constituents as potential solvents for CO_2_ capture. However, melting properties of pure constituents influence the melting temperature of the DES (Alhadid et al., 2019). M236 and M246 in 1:2 ratio are solid at room temperature, which is attributed to the high melting temperature of 2,3,6- and 2,4,6- trimethylphenols (T_m_ = 331.2 and 342.15 K, respectively) (Verevkin, 1999). MTH and M26X in 1:2 ratio are liquid at room temperature (Alhadid et al., 2021a), which indicates that both can be considered for further experimental investigation. CO_2_ activity coefficients in MTH and M26X at 1:2 ratio are of similar values (Figure 2). However, MTH is considered a better option because of the low toxicity of thymol compared to 2,6-xylenol. Therefore, MTH was chosen for measurements.

Next, eutectic mixtures containing two phenolic alcohols were considered. These eutectic mixtures are expected to show ideal solution behavior with no significant decrease in the melting temperature of the mixture relative to pure constituents (Alhadid et al., 2019). For such mixtures, the melting temperature of the DES at any ratio between constituents can be obtained from the solid–liquid phase diagram based on the pure constituent melting properties. A brief explanation of solid–liquid equilibrium calculations is given in the Supplementary Material. Based on the ideal solution model calculations, thymol: 2,6-xylenol (T26X) eutectic system should form a liquid mixture at room temperature. The calculated eutectic composition and temperature for T26X are x_e,thymol_ = 0.46, and T_e_ = 292.8 K, respectively (see Supplementary Material for details about the calculations). Altering the ratio between constituents in T26X does not influence CO_2_ activity coefficients (Figure 2), in contrast to what is observed in L-menthol-based DES. Thus, the molar ratio close to the eutectic ratio of the T26X system (∼1:1 ratio) was selected to ensure that the mixture is liquid at room temperature. Eventually, the two DES, MTH in 1:2 ratio, and T26X in 1:1 ratio were selected for the solubilty measurements.

### Experimental Solubility

CO_2_ solubility in the two DES was measured based on the pressure-drop isochoric method at four different pressures (∼4, 3, 2, and 1 MPa) and four different temperatures (293.15, 303.15, 313.15, and 323.15 K). The CO_2_ solubility in weight percentage $\left(w_{CO_{2}}\right)$ and mole fraction $\left(x_{CO_{2}}\right)$ , as well as its fugacity coefficient $\left(\phi_{{\text{CO}}_{2}}\right)$ calculated using Span and Wagner equation of state for the MTH and T26X system are shown in Tables 3, 4, respectively. The values of Henry’s constant at different temperatures calculated using Eq. 2 are shown in Table 5. As one would expect, the solubility of CO_2_ in the two studied systems decreases as temperature increases.

**TABLE 3:** Experimental carbon dioxide (CO_2_) solubility (both in weight percent $w_{CO_{2}}$ and in mole fraction $x_{CO_{2}}$ ) in the L-menthol/thymol (MTH) eutectic system and calculated CO_2_ fugacity coefficient $\left(\phi_{CO_{2}}\right)$ at various temperatures T and pressures p.^a^

*T*/K	*p*/MPa	$w_{CO_{2}},$ %	$x_{CO_{2}}$ /Mole fraction	$\phi_{CO_{2}}$ ^b^
323.11	4.1939	11.410	0.3082	0.8453
313.12	4.1543	12.090	0.3224	0.8295
303.18	4.0967	13.409	0.3488	0.8121
293.15	4.0201	15.769	0.3931	0.7931
323.15	3.5059	9.290	0.2616	0.8701
313.16	3.4703	10.173	0.2815	0.8570
303.13	3.4247	11.780	0.3159	0.8426
293.13	3.3691	13.335	0.3474	0.8265
323.14	2.5790	7.090	0.2088	0.9039
313.18	2.5541	7.615	0.2219	0.8942
303.13	2.5229	8.347	0.2395	0.8835
293.14	2.4860	9.297	0.2617	0.8716
323.02	1.6819	4.644	0.1442	0.9369
313.18	1.6662	5.027	0.1547	0.9305
302.98	1.6478	5.436	0.1659	0.9235
293.15	1.6281	5.937	0.1792	0.9155

a Standard uncertainties *u* are: *u*(*T*) = 0.030 K, *u*(*p*) = 0.1%, *u*(*w*) = 0.01 wt%, and *u*(*x*) = 0.0001 mol fraction.

b Calculated using the Span and Wagner equation of state (Span and Wagner, 1996) implemented in ThermoFluids (Springer, V. 1.0).

**TABLE 4:** Experimental carbon dioxide (CO_2_) solubility (both in weight percent $w_{CO_{2}}$ and in mole fraction $x_{CO_{2}}$ ) in the thymol/2,6-xylenol (T26X) eutectic system and CO_2_ calculated fugacity coefficient $\left(\phi_{CO_{2}}\right)$ at various temperatures T and pressures p.^a^

*T*/K	*p*/MPa	$w_{CO_{2}},$ %	$x_{CO_{2}}$ /Mole fraction	$\phi_{CO_{2}}$ ^b^
322.90	4.0853	11.549	0.2878	0.8492
313.13	4.0441	12.563	0.3078	0.8339
303.17	3.9840	14.856	0.3506	0.8172
293.22	3.9057	17.093	0.3895	0.7990
323.14	3.3380	9.512	0.2455	0.8762
313.15	3.3029	10.417	0.2646	0.8638
303.13	3.2563	11.456	0.2859	0.8502
293.14	3.1988	13.073	0.3176	0.8352
323.13	2.6988	7.477	0.2000	0.8995
313.15	2.6696	8.022	0.2125	0.8895
303.16	2.6344	9.205	0.2388	0.8784
293.15	2.5932	10.284	0.2619	0.8661
323.14	1.6670	4.462	0.1263	0.9375
313.15	1.6500	4.909	0.1377	0.9313
303.16	1.6300	5.398	0.1501	0.9243
293.15	1.6070	6.057	0.1663	0.9166

a Standard uncertainties *u* are: *u*(*T*) = 0.030 K, *u*(*p*) = 0.1%, *u*(*w*) = 0.01 wt%, and *u*(*x*) = 0.0001 mol fraction.

b Calculated using the Span and Wagner equation of state (Span and Wagner, 1996) implemented in ThermoFluids (Springer, V. 1.0).

**TABLE 5:** Calculated carbon dioxide Henry’s constant $\left(H_{CO_{2}}\right)$ in the studied systems.

T/K	$H_{CO_{2}}$ /MPa	
	L-menthol/thymol (1:2)	Thymol/2,6-xylenol (1:1)
293.15	8.52 ± 0.13	9.45 ± 0.32
303.15	8.98 ± 0.13	10.45 ± 0.23
313.15	9.61 ± 0.25	11.46 ± 0.15
323.15	10.51 ± 0.30	12.52 ± 0.17

**FIGURE 3:**
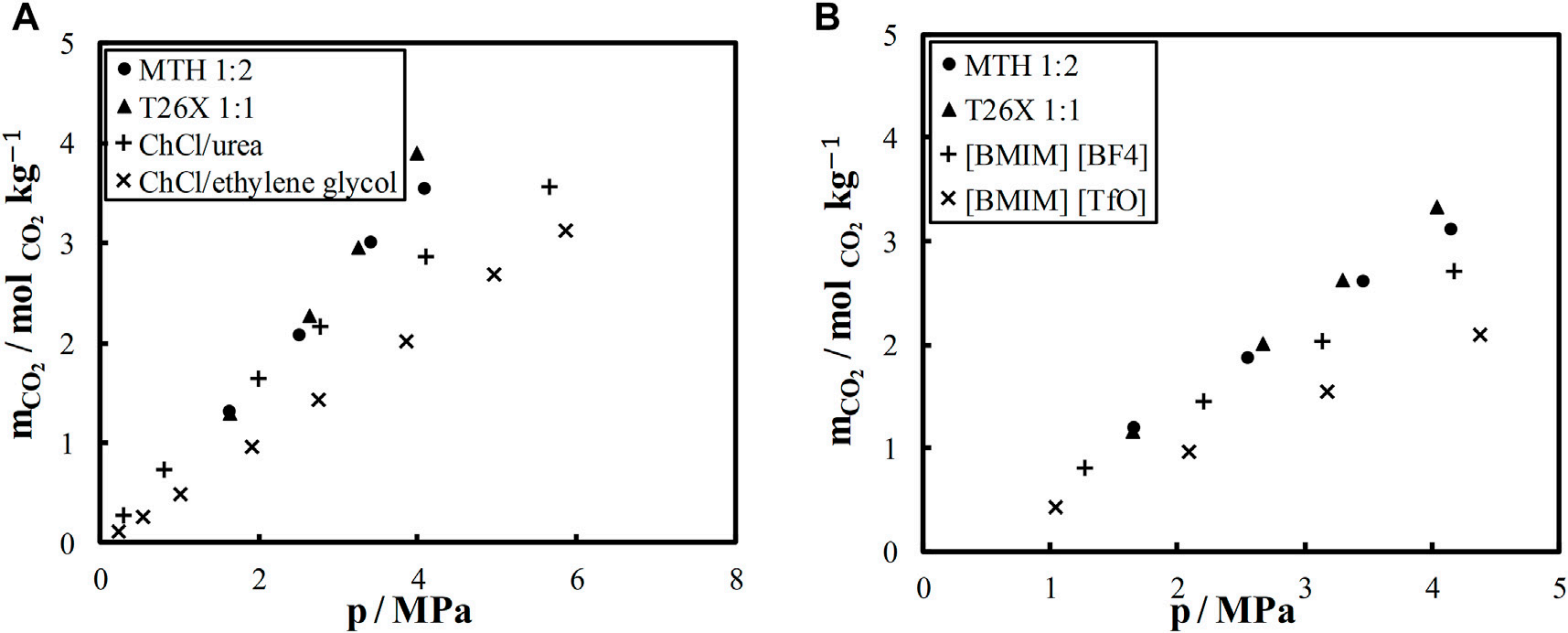
Carbon dioxide solubility **(A)** in L-menthol/thymol (MTH), thymol/2,6-xylenol (T26X), choline chloride (ChCl)/urea (Leron et al., 2013), and ChCl/ethylene glycol (Leron and Li, 2013) at 303.15 K **(B)** and in MTH, T26X (BIMI) (BF4), and (BMIM) (TfO) (Aki et al., 2004) at 313.15 K.

Further, the CO_2_ solubility in the studied DES was compared with that in some ionic DES and IL reported in the literature. The comparison was made in terms of molality, i.e., moles of CO_2_ absorbed per mass of solvent. The results are shown in Figure 3. CO_2_ solubility at 303.15 K in two ionic DES, namely, choline chloride (ChCl)/urea and ChCl/ethylene glycol in 1:2 molar ratio, is compared with the two DES from this study in Figure 3A. As seen, CO_2_ solubility is significantly higher in nonionic DES than in ionic DES, especially at high pressures (Figure 3A). The CO_2_ solubility in MTH and T26X is also higher than in the two IL (BMIM) (BF4) and (BMIM) (TfO), as shown in Figure 3B. In addition to good CO_2_ solubility, nonionic DES are more stable, less hygroscopic, and less expensive than IL and ionic DES.

**FIGURE 4:**
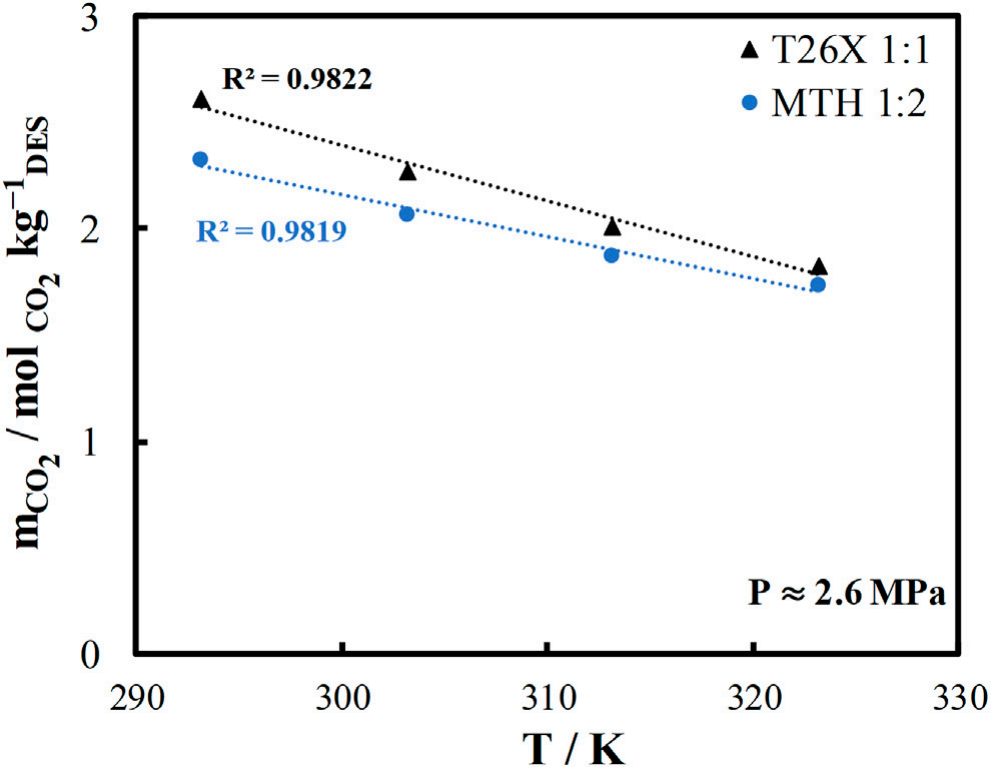
Temperature dependence of carbon dioxide solubility at medium pressure ∼2.6 MPa in L-menthol/thymol (MTH) and thymol/2,6-xylenol (T26X).

The solvent capacity to absorb CO_2_ is not the only selection criterion to consider when selecting a solvent for CO_2_ capture applications. The temperature dependence of CO_2_ solubility is critical as well because CO_2_ should be absorbed with high solubility and desorbed from the solvent at a higher temperature for solvent regeneration. Thus, a strong temperature dependence is required for CO_2_ solubility to reduce the energy demand for desorption. The temperature dependence of CO_2_ solubility in the two DES being studied is shown in Figure 4. The T26X system shows a slightly higher CO_2_ solubility than the MTH system. Furthermore, the T26X system has a stronger temperature dependence. Therefore, the T26X system can be identified as a very promising candidate for CO_2_ capture applications.

## Conclusion

This study examines the use of nonionic DES for CO_2_ capture applications. DES were designed to contain phenolic alcohols for improving CO_2_ solubility and L-menthol to decrease the melting temperature of the DES. COSMO-RS was used to preselect the DES constituents from a pool of possible phenolic alcohols and to tune the molar ratio between the constituents. It was found that the structure of phenolic alcohols can influence CO_2_ solubility. Furthermore, increasing the phenolic alcohol molar content in the DES can enhance the CO_2_ solubility. However, the selection of the constituents and their molar ratio was restricted by the melting temperature of the DES. The COSMO-RS screening results identified two potential DES: MTH in 1:2 molar ratio and T26X in 1:1 molar ratio.

In the two preselected DES, the CO_2_ solubility was studied experimentally using a pressure-drop isochoric method at various temperatures and pressures. The high experimentally determined CO_2_ solubility in the two DES validated the COSMO-RS preselection, demonstrating the model’s advantage as a screening tool. CO_2_ solubility in the DES proposed in this study is significantly higher than in ionic DES and IL proposed in the literature. Furthermore, the temperature dependence of CO_2_ solubility in DES proves their suitability for CO_2_ capture applications. The high solubility of CO_2_ at lower temperatures and its high decrease at higher temperatures make the two DES promising candidates for CO_2_ capture. This study shows that simple and widely available organic substances can be used to form novel solvents with unique properties.

## Data Availability

The original contributions presented in the study are included in the article/Supplementary Material, further inquiries can be directed to the corresponding authors.
