# Supplementary material for: Carbon Dioxide Solubility in Nonionic Deep Eutectic Solvents Containing Phenolic Alcohols
Source: Front Chem. 2022 Mar 22;10:864663. doi: 10.3389/fchem.2022.864663 (PMC8980276; doi:10.3389/fchem.2022.864663)
Supplement: Supplementary file 1 [file Table1.DOCX]

Supplementary Material

Table S1. Vapor pressure of pure constituents calculated using the Antoine equation. (Stull, 1947)

| T / K | $p^{sat}$ / mbar | | |
| --- | --- | --- | --- |
|  | L-menthol | thymol | 2,6-xylenol |
| 293.15 | 0.0911 | 0.0576 | 0.2291 |
| 298.15 | 0.1382 | 0.0865 | 0.3496 |
| 303.15 | 0.2063 | 0.1281 | 0.5234 |
| 308.15 | 0.3036 | 0.1870 | 0.7695 |
| 313.15 | 0.4406 | 0.2694 | 1.1125 |
| 318.15 | 0.6308 | 0.3833 | 1.5832 |
| 323.15 | 0.8920 | 0.5387 | 2.2200 |
| 328.15 | 1.2462 | 0.7486 | 3.0700 |
| 333.15 | 1.7216 | 1.0291 | 4.1906 |
| 338.15 | 2.3528 | 1.4003 | 5.6504 |
| 343.15 | 3.1827 | 1.8868 | 7.5312 |
| 348.15 | 4.2637 | 2.5187 | 9.9294 |
| 353.15 | 5.6590 | 3.3325 | 12.9572 |

**References**

Stull, D.R. (1947). Vapor Pressure of Pure Substances. Organic and Inorganic Compounds. *Industrial & Engineering Chemistry* 39(4)**,** 517-540. doi: 10.1021/ie50448a022.
