# Supplementary material for: Carbon Dioxide Solubility in Nonionic Deep Eutectic Solvents Containing Phenolic Alcohols
Source: Front Chem. 2022 Mar 22;10:864663. doi: 10.3389/fchem.2022.864663 (PMC8980276; doi:10.3389/fchem.2022.864663)
Supplement: Supplementary file 2 [file DataSheet1.DOCX]

Supplementary Material

**Ideal solution model**

The considered DES should be liquid in the proper temperature range suitable for carbon dioxide capture. Solid–liquid phase diagram can be used to determine the melting temperature of the mixture at any composition. Most studied DESs have been considered as simple eutectic systems in which pure constituents crystallize in the pure solid form. Figure S1 shows a schematic representation of a solid–liquid phase diagram of a simple eutectic system. The blue curves are the liquidus line of Components A and B. The liquidus lines intercept at the eutectic point, which corresponds to the lowest melting temperature of the mixture with the eutectic composition.

**Figure 1**. Schematic representation of a solid–liquid phase diagram of a simple eutectic system

The liquidus lines can be calculated as follows (Prausnitz et al., 1999)

| $\ln x_{i}\gamma_{i}=-\frac{{\Delta h}_{m,i}}{RT}\left( 1-\frac{T}{T_{m,i}} \right)$ | (1) |
| --- | --- |

where $x_{i}$and $\gamma_{i}$ are the mole fraction and activity coefficient of component $i$ in the liquid phase; ${\Delta h}_{m,i}$ and $T_{m,i}$ are the melting enthalpy and the melting temperature of component $i$; T is the liquidus temperature, and R is the universal gas constant. When the solution is assumed as ideal ($\gamma_{i}=1$), the phase diagram can be modeled using only the melting properties of the pure components as follows

| $\ln x_{i}^{L}=-\frac{{\Delta h}_{m,i}}{RT}\left( 1-\frac{T}{T_{m,i}} \right)$ | (2) |
| --- | --- |

According to Eq. (2), the position of the eutectic point and the whole SLE phase diagram of an ideal eutectic system depend on the melting properties of the pure components. For the thymol/2,6-xylenol eutectic system, the eutectic point was determined using Equation (2) with the melting properties shown in Table S2.

Table S2. Melting properties of pure components.

| Substance | T_m_ /K | Δh_m_ /kJ mol^–1^ |
| --- | --- | --- |
| thymol | 322.7 (Alhadid et al., 2020) | 20.64 (Alhadid et al., 2020) |
| 2,6-xylenol | 318.6 (Jamróz et al., 1998) | 18.83 (Jamróz et al., 1998) |

**References**

Alhadid, A., Mokrushina, L., and Minceva, M. (2020). Formation of glassy phases and polymorphism in deep eutectic solvents. *Journal of Molecular Liquids* 314**,** 113667. doi: <https://doi.org/10.1016/j.molliq.2020.113667>.

Jamróz, M.E., Palczewska-Tulińska, M., Wyrzykowska-Stankiewicz, D., Szafrański, A.M., Polaczek, J., Dobrowolski, J.C., et al. (1998). The urea–phenol(s) systems1Paper presented at the International Conference on Applied Physical Chemistry, Warsaw, 13–15 November 1996.1. *Fluid Phase Equilibria* 152(2)**,** 307-326. doi: <https://doi.org/10.1016/S0378-3812(98)90206-0>.

Prausnitz, J.M., Lichtenthaler, R.N., and Azevedo, E.G.d. (1999). *Molecular Thermodynamics of Fluid-Phase Equilibria.* Upper Saddle River, NJ: Prentice Hall PTR.
